# Model transcriptional networks with continuously varying expression levels

**DOI:** 10.1186/1471-2148-11-363

**Published:** 2011-12-19

**Authors:** Mauricio O Carneiro, Clifford H Taubes, Daniel L Hartl

**Affiliations:** 1 Department of Organismic and Evolutionary Biology, Harvard University, Cambridge, MA, USA, 02138; 2 Department of Mathematics, Harvard University, Cambridge, MA, USA, 02138

## Abstract

**Background:**

At a time when genomes are being sequenced by the hundreds, much attention has shifted from identifying genes and phenotypes to understanding the networks of interactions among genes. We developed a gene network developmental model expanding on previous models of transcription regulatory networks. In our model, each network is described by a matrix representing the interactions between transcription factors, and a vector of continuous values representing the transcription factor expression in an individual.

**Results:**

In this work we used the gene network model to look at the impact of mating as well as insertions and deletions of genes in the evolution of complexity of these networks. We found that the natural process of diploid mating increases the likelihood of maintaining complexity, especially in higher order networks (more than 10 genes). We also show that gene insertion is a very efficient way to add more genes to a network as it provides a much higher chance of developmental stability.

**Conclusions:**

The continuous model affords a more complete view of the evolution of interacting genes. The notion of a continuous output vector also incorporates the reality of gene networks and graded concentrations of gene products.

## Background

In the approximately ten years since the completion of the draft sequence of the human genome, researchers have become increasingly attuned to the many layers of complexity that underlie the mechanisms of life [1]. Many new genes have been identified for transcription factors whose role is to activate or inhibit the production of other genes. The interplay between mutually interacting transcription factors defines a regulatory network that dictates the levels of RNA transcripts, signaling proteins, enzymes and other gene products. Such networks have emergent properties that are essential in every living system[2].

Understanding the organization and evolution of these networks has been a challenge because of their complexity. Experimental studies have been able to identify important roles of interacting regulatory networks, such as the ability of yeast to respond to environmental changes[3], the specification of the endomesoderm in sea urchin embryos[4], and dorsal-ventral patterning in the *Drosophila *embryo[5]. Although early studies of quantitative traits also revealed clues about such networks[6], it has not been generally feasible to address the more general questions how they originate and evolve.

A mathematical model of mutually interacting transcription factors was first developed by Wagner[7]. In this model, the level of expression of *n *transcription factors in an individual at time *t *is given by the values of the elements in a vector S with *n *entries. The mutual interactions between transcription factors are represented as an *n *× *n *matrix *W *whose elements *w_ij _*are real numbers. W defines the gene network, and the distribution of the non-zero entries of *W *specifies how connected the network is. Each row in *W *corresponds to a single transcription factor, and *W_ij _*represents the effect of transcription factor *j *on the production of transcription factor *i*. The vector *S_t _*of expression levels in an individual changes in ontology according to a characteristic time constant τ according to *S_t+τ _*= *f*(*W *× *S_t_*), where *f *is a realvalued function applied element by element to the entries of *W *× *S_t_*. In Wagner's model[8], *f *(*s_i_*) = -1, 0, or +1 according to whether the *i*-th element *s_i _*of *W *× *S_t _*is < 0, = 0 or > 0. The model therefore allows gene expression to be either completely repressed (-1), expressed at a basal level (0), or completely derepressed (+1). An individual is characterized by the final contents of its gene expression vector S. An individual is considered *viable *if, and only if, the final gene expression level vector converges to a stable state, meaning, even if the developmental process were continued [*S_t+τ _*^= ^*f*(*W *× *S_t_*)] *S *would remain unchanged. Occasionally we use the term *viable network*, by which we mean a *W *matrix capable of yielding viable individuals from a subset of initial state vectors.

The gene expression patterns generated by this model were able to mimic certain aspects of *Drosophila *gene expression data [8] However, the Wagner model does not allow for differing concentrations of the transcription factors. Further studies [9]were stimulated by the potential of Wagner's original model. Siegal and Bergman[9] adapted the model to simulate the evolution of such transcriptional networks, which revealed that stabilizing selection in a population is sufficient to evolve robustness as predicted by Waddington's canalization model of development[10]. In their model, Siegal and Bergman included parameters to dictate the shape of a sigmoidal function, which would allow for a continuous model to be explored, however in all their simulations the parameters were chosen such that the output vector would behave exactly like Wagner's.

This work was followed by additional studies focusing on such issues as network robustness to mutation[11-14]. Bergman and Siegal [15]showed that knockout mutations (replacing an entire row and column with zeroes) would significantly increase the sensitivity to initial conditions, but later studies showed that the relationship between a gene's connectivity and its fitness effect upon knockout depends on its equilibrium expression level[16]. In all these studies, the model was used with the limitation of Wagner's original -1,0,1 output vector. In the model discussed in detail below, we propose an alternate definition of deleterious mutation and analyze its effect on viability in a continuous output scenario.

Sexual and asexual reproduction as well as the coevolution of reproductive method and genetic architecture were analyzed by Azevedo *et al*[17,18]. They showed that sexual reproduction evolves greater robustness than asexual reproduction, using the same canalization[10] framework as Siegal and Bergman[9]. But both studies rely on a version of the model in which transcription factors are either on or off and all mating is treated as haploid. In this study we show that, under the continuous model, the effects of mating in haploid and diploid populations are quite different.

The network structure of the Wagner model has been studied in many ways[7,8,13,19-22], and the results suggest that large networks evolve to be sparse[16,20,23,24] and modular[2,21,25,26], The general argument holds that the cost of maintaining unnecessarily complex interactions is too large to be maintained[20], and once modules of interactions (i.e. smaller networks that achieve a viable final state) are formed, it is easier to combine them together than to evolve the same mechanisms *de novo *in new networks [21].

Other types of network models have also been developed. A system of coupled ordinary differential equations derived from the principle of chemical kinetics was used to describe genes and the concentration of their products[27]. Boolean networks were also used to illustrate how gene interaction networks could be modeled together with their products assuming just two states, on or off[24,28,29]. Stochastic models[30] based on the Gillespie algorithm[31,32,31]were motivated by recent experimental results that have demonstrated that gene expression can have a component affected by stochastic noise[33-35]. These models are limited to very small networks due to the inherent complexity of the algorithm[36,37].

A continuous network model (that is, one in which the elements *w_ij _*in *W *and *s_j _*in the vector S are continuous real variables) might be capable of describing properties of the regulatory networks not present in the discrete model. One approach to a model of a regulatory network with continuous output was based on using artificial neural networks[38] to describe cellular differentiation and morphogenesis[39].

The discrete version of the Siegal and Bergman[9] model is limited by the concept of viability. Because of the bounding of the gene expression levels to discrete values (-1, 1), the development process *S*_*t*+1 _= *f*(*W *× *S_t_*) will discard every network in which the matrix *W *does not have an eigenvector pointing towards one of the vertices of the hypercube centered at the origin. In this sense most viable individuals in a population created using the discrete model will be essentially similar. In a continuous model, networks that have eigenvectors of the matrix *W *pointing to an edge or face of the hypercube can also yield viable networks. This is due to the broader range of acceptable values of the output vector. As a consequence, the continuous model should yield a more diverse population of developmentally stable matrices (a formal analysis of this point is presented in the additional file 1).

In this work, we describe another approach to a network model with continuous output generalizing that in Wagner (1996). The continuous variation in the abundance of the gene products creates additional complexity that allows a more complete description of the evolution of these networks. The model is intuitively appealing because different concentrations of transcription factors should affect gene expression quantitatively, resulting in different levels of activation and repression. The output vector would therefore be expected to be composed of elements that are continuous rather than discrete. We build on Wagner's model[8] in order to allow detailed comparison between the continuous and discrete versions of the model.

## Methods

Our model is similar to that of Siegal and Bergman[9] in positing a gene regulatory network of *n *genes represented by the *n *× *n *matrix *W*, where *w_ij _*measures the extent to which the abundance of the product of gene *j *affects the production of the product of gene *i*. Given a vector *S_0 _*of initial expression levels in an individual for each of the *n *genes, the individual is tested for "viability" in a stepwise process of "development" [*S*_*t*+1 _= *f*(*W *× *S_t_*)] that tracks the amounts of the transcription factors in the individual over time. A viable individual is an individual that develops a stable output vector *S*.

The concept of stability is based on the development of the individual represented at time *t *by the vector *S_t_*. Each developmental step is modeled as the result of multiplication between the matrix *W *and the vector *S_t_*, yielding a new vector *S_t+1_*, which is multiplied again by the same matrix *W *until the variation in *S_t+1 _*is less than some sensitivity constant *σ *when compared to previous values.

The multiplication of each row *i *of the matrix *W *by the vector *S *represents the interaction between every gene in the network. Each value in the matrix describes the type and strength of the interaction. Positive values mean activation or enhancement of production, whereas negative values mean repression or inhibition. Because the values in the matrix are continuous in the interval [-1, 1], the absolute value describes the strength of the interaction, be it positive or negative.

By multiplying *W *with the vector of gene products, we scale the effects of the direct and indirect interactions, adding together the weighted effect of every interaction between each gene and every other gene in the network. The result is an updated vector calculated as:

(1) $$S_{t+1}=f\left(\overset{N}{\underset{j=1}{\sum }}w_{ij}s_{j\left(t\right)}\right)$$

where the function *f *is as described below. Each new *S_t+1 _*is evaluated by a measure analogous to the variance (*D*) against the mean of the last τ (in this work τ = 10 for all simulations) state vectors obtained in previous iterations. If φ (defined below) is less than a sensitivity margin σ, then the vector *S_t _*is considered stable and its final state designated *S*,

(2) $$\varphi \left(S_{t}\right)=\frac{1}{\tau }\overset{t}{\underset{i=t-\tau }{\sum }}D\left(S_{i},{\bar{S}}_{t}\right)$$

where $D\left(X,Y\right)=\overset{N}{\underset{i=1}{\sum }}{\left(X_{i}-Y_{i}\right)}^{2}∕\left(4N\right)$lies in the interval [0, 1] and ${\bar{S}}_{t}$ is the average of the expression levels over all times from *τ*- *t *to *t*. The number of genes in the network is *n*, and *X *and *Y *are vectors with *n *elements each. The number of steps (*L*) to reach stability is taken as a measure of path length. *L_max _*= 100 is the cutoff value in the number of development steps, after which a matrix is considered unstable. As a stability criterion we chose σ = 10^-4^. This model yields viable individuals in which the levels of the transcription factors are continuous, and is a straightforward extension of Wagner's model of transcription regulatory networks[8,14]. Both models discard networks with imaginary eigenvalues of the matrix *W *owing to the cyclic behavior of the output vector and the lack of convergence to stable value of *S*.

If one were to define *S*_*t*+1 _merely as *S*_*t+*1 _= *W *× *S_t_*, the expression levels in *S_t _*could grow to positive or negative infinity unless constrained. To constrain these values we follow Siegal and Bergman[9] in applying a scaling function (*f*) that bounds the values of the upper and lower limits of gene production to 1 and -1. This function plays the role of confining the expression levels to the interval [-1, 1] in a potentially continuous fashion. Specifically,

(3) $$f\left(x\right)=\frac{2}{\left(1+e^{-ax}\right)}-1$$

which is a sigmoidal function centered at *x = 0*. Its curvature is determined by the constant *a*. Although the bounding effect of this function is obvious, the extent to which it spreads the expression levels is subtler and depends on the curvature.

We analyzed different values of *a*, and found that values greater than 2 result in a very large variance, forcing all values of the final state vector close to either -1 or 1, essentially making it a discrete model. Between the values of 1 and 2, however, we found that the values of the final state vector would be well distributed along the -1, 1 interval allowing us to use the model in a continuous fashion without affecting the number of viable networks we can yield through our development process. Hence for all simulations in this work, we used *a *= 1.5. This choice of *a *is virtually identical to the sigmoidal *tanh *function used by Kaneko (2011)[40]

The continuous model gives us a more complete view of the evolution of interacting genes. It allows the addition of more genes to the networks and is more efficient in maintaining stability. The notion of a continuous output vector also creates a closer relationship with the reality of gene networks and gene products, where it is not sufficient to ascertain merely whether a gene is on or off. Gene product concentrations play an important role in determining the viability of individuals, and aids in the evolution and maintenance of complexity.

The different mechanisms to generate network complexity tested had a strong impact in the probability of yielding a viable individual, even in networks with many genes and especially for diploid mating. By "diploid mating", we mean that each element of the matrix W of the progeny network equals the arithmetic average of the corresponding elements in the parental matrices. The probability that a random network of 15 genes yields a viable individual is far smaller than that obtained by diploid mating between two viable networks suggests that sexual reproduction may be a key component in the evolution of complexity. The phenomena of insertion and deletion probably also play an important role in the evolution of complexity, given the high probability of a viable individual to remain viable after undergoing an insertion or a deletion.

The continuous model also gives insight into the mechanisms that regulate the evolution of complexity in a general setting that represents the concentration of the products of gene networks. The inclusion of gene product concentration brings the model closer to actual transcriptional networks, and perhaps gives us a better idea of the difficulty of obtaining complex networks that yield viable individuals in the real world.

## Results and Discussion

### Intraclass Correlation

The final stable output vector describes the gene expression levels of a viable individual. The distribution of output values on (-1, +1) across individuals was indistinguishable from a uniform distribution, as might be expected. We also tested whether the output vectors were correlated across individuals. To test for correlation, we examined the intra-class correlation coefficient (ICC) of the elements of *n *stable output vectors, each from a distinct viable network with *k *genes:

(4) $$ICC=\frac{1}{n-1}\left[\frac{n}{ks^{2}}\overset{k}{\underset{i=1}{\sum }}{({\bar{x}}_{k}-\bar{x})}^{2}-1\right]$$

The ICC tests whether the final output vectors of the *n *viable individuals are clustered together in small regions of the space [-1, 1]. In the equation for ICC, ${\bar{x}}_{n}$ is the sample mean of the elements in the *n*-th individual, $\bar{x}$ is the mean of all output vectors in the population, *k *is the number of genes in the network, and *s*^2 ^is the variance of the elements among the *n *individuals. The value of ICC can be positive or negative, but a value close to zero means not correlated, whereas a value close to 1 or -1 means high intra-class correlation.

**Figure 1:**
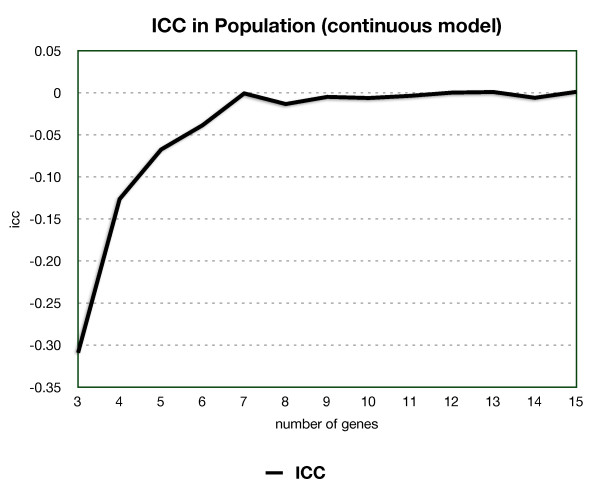
**The intraclass correlation coefficient (ICC) indicates how closely gene products levels are clustered in viable individuals**. An ICC of 0 means uncorrelated.

From our data for both the discrete and continuous models, for any number of genes, we found the ICC value very close to zero. Even with a small number of genes where there is little room for variation, the ICC was still extremely low, as shown in Figure 1 for the continuous model.

### Generating Viable Individuals

Individuals were generated at random by drawing networks and initial vectors of gene expression levels from a uniform distribution on [-1, +1]. One possible interpretation of the initial vector is that it is the level of gene products passed by maternal inheritance to the zygote, where development of the embryo would begin.

**Figure 2:**
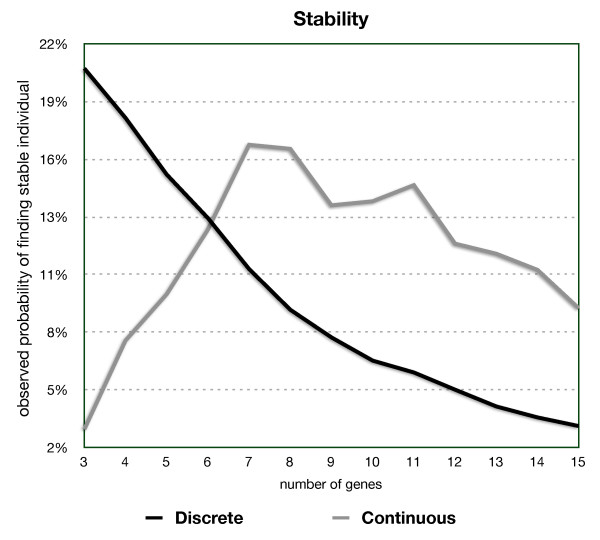
**Likelihood of finding a viable individual with a random input vector in the discrete and continuous models**. The Discrete Output Vector model (DOV) is an adaptation of Wagner's original model with continuous values in the network and discrete values in the output vector, resulting from a choice of *a *= 100 in Equation 3. The additional requirements for stability still hold (minimum population variance of 0.1). The continuous output vector model (COV) rarely yields viable individuals in networks with a small number of genes, but quickly matches and exceeds the likelihood of the DOV in networks with 6 genes. With more than 7 genes, the COV is actually more efficient at yielding viable individuals than the DOV, while maintaining a higher population variance (not shown).

We generated 12 populations of 1000 viable individuals each (one population for each network from 3-15 genes). Figure 2 shows the likelihood of finding a viable network at random. It is clear that viable networks with many genes are unlikely to occur by chance.

The model depends on a set of initial conditions to start the developmental stage of the simulation using a randomly generated initial state vector. It is therefore unclear whether the viability of these individuals is determined by the choice of the initial output vector or by the wiring of the gene network.

To test the impact of the choice of an initial output vector we selected viable individuals and replaced their networks with randomly generated ones, however retaining the original initial state vector. We repeated this process 1000 times for each individual, generating a different network each time, while tallying the number of random *W *matrices that supported development into a viable individual. Analogously, we performed a similar test by keeping the network while randomly changing the initial state vector instead. These tests enabled an analysis of the relation between stability due to the input vector and stability due to the network.

**Figure 3:**
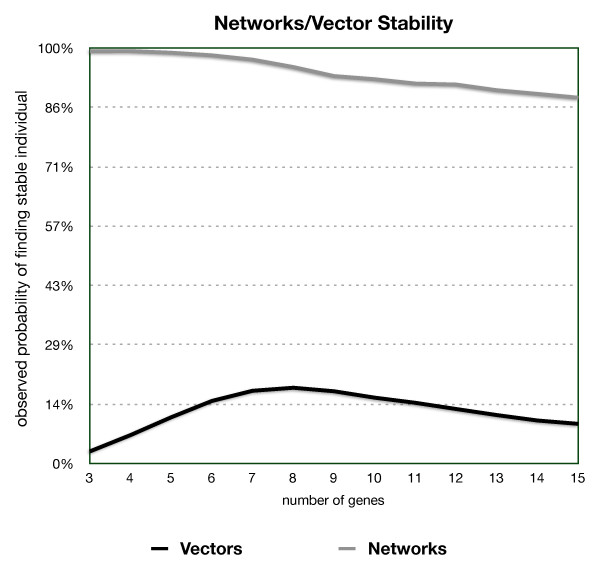
**Frequency of viable individuals generated from a viable network and from a viable initial state vector**.

As shown in Figure 3, at most 20% of the vectors tested for any number of genes in the network were responsible for stability, yielding numbers very close to those of viabilities of initial state vectors drawn at random; therefore, the initial state vector has little or no effect on generating viable individuals. The network itself, however, is highly correlated with viability. In the discrete output model, viability was determined by the choice of matrix and averaged about 70% of the 12 million tests.

### Evolution of Complexity

Given the very low likelihood that a random *W *matrix with a high number of genes will be viable (that is, support development of random vectors to a stable state as defined by Equation [2]), we tested how easily complexity might evolve from combining ("mating") viable networks and producing a new network that may be interpreted as the "offspring" of two viable networks.

Mating was performed by defining a population of 1000 viable networks and mating two randomly drawn networks at a time. The "offspring" network was then tested for stability by iterating random initial vectors according to Equation 1. This process was repeated 1000 times to generate 1000 new progeny networks.

### Haploid Mating

In the process of "haploid mating", a given gene is inherited at random from the network of either parent with equal probability. Accordingly, in the haploid mating process, we randomly selected individual rows from within the paternal or maternal network and copied them to create an offspring network. This process passes on parental genes without modification from one generation to the next. Repeating the selection process for each row yields a new offspring network with a random set of both parents' genes.

The initial state vector of the new offspring is chosen at random to equal a stable state of one of the parents. This procedure reflects the assumption that one of the parents would be passing on the general stable gene-product concentrations to its offspring, analogous to the interaction between an oocyte and its mother during the earliest stages of development.

**Figure 4:**
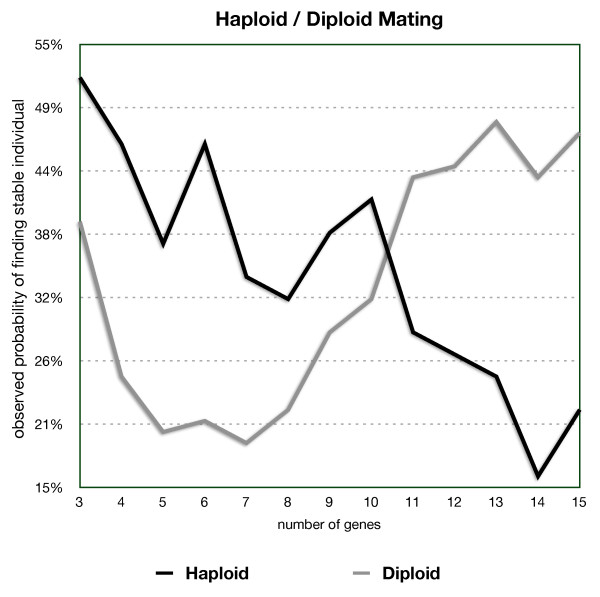
**Haploid and diploid mating stability**. When compared with the DOV model, diploid mating displays a very different behavior. As with the haploid model, the discrete model excels in smaller networks, with rates as high as 60%, but falls sharply and starts oscillating between 10-40% in individuals with more than 10 genes. The continuous model shows the opposite pattern starting at values between 20-40% for small networks and consistently increasing as the number of genes grows, achieving values of 43-47% for the rate of viability with networks greater than 10 interacting genes. This result suggests that diploid mating has a greater impact on viability in the continuous model.

When applied to a population of 1000 viable networks (see Figure 4), haploid mating maintained a stability rate higher than 40% for progeny networks with up to 6 genes. The stability rate drops, however, to 30-40% in networks with 7 to 10 genes, and drops further to between 20-30% for networks with more than 10 genes. This result suggests that it is possible to generate complex networks with haploid mating with a much higher likelihood than generating them at random. Haploid mating is especially efficient at maintaining network stability for lower complexity networks.

It is interesting to draw a parallel between haploid mating in the discrete model and the continuous one. Haploid mating displays the same behavior in both models, with high efficiency in generating viable networks with a small number of interacting genes, but then efficiency falls off sharply as the number of genes increase. In the case of the discrete model, the efficiency drops to almost zero with 8 or more genes. In contrast, the continuous model maintains a more consistently slower drop with increasing number of genes, without ever reaching 0 even for networks of size 15.

### Diploid Mating

Diploid individuals benefit from heterozygosity to modulate the effects of damage or deleterious mutations as well as from increasing diversity through the recombination events between the parental chromosomes. In the process of "diploid mating," each row in the *W *matrix of the progeny is calculated as the arithmetic mean of the corresponding rows in the *W *matrices of the parents. Biologically, this means that the effects on gene expression are additive, and effects due to dominance, overdominance, underdominance, epigenetics, parent of origin, and so forth are ignored. Taking the impact of each gene as the average of the impacts of this same gene in each parent tends to mitigate large negative or positive effects of the parental genes.

When applied to a set of 1000 viable networks, the diploid mating model generated viable progeny networks of up to 10 interacting genes in 19-32% of the iterations (Figure 4). This percentage is not as high as that in the haploid model, but diploid mating performs better as the number of genes increases. For networks with more than 10 genes, the number of viable offspring networks lies between 43-48%. The positive slope of the curve shows that the diploid mechanism with additive gene effects is very efficient in maintaining stability in complex networks.

A randomly generated network with 15 interacting genes has an 8.9% chance of being viable. When two viable individuals mate following the haploid-mating model, the likelihood of generating a viable network jumps to 22%, however diploid mating increases the likelihood to 47%. This increase may be due to the fact that these original two networks were already selected from a small pool of viable networks with 15 genes, and diploid mating maintains network stability better than haploid mating. We conclude that, while for any level of complexity (number of genes in the network) it is difficult to generate viable complex individuals at random, mating is relatively efficient in producing viable networks of the same level of complexity as those in the parents.

### Random Insertion

The difficulty in finding a viable network with more than 10 interacting genes prompted the question of whether increasing the number of genes of a viable network is more successful than generating a viable network at random. To answer this question we randomly inserted a gene into a viable network and developed stable state vectors to test whether stability was retained.

A gene insertion represents the phenomenon of a new gene being fully incorporated by the genome and interacting with the other genes in the network. In the inserted gene all interaction values are chosen at random from the uniform distribution [-1,1], and all pre-existing genes receive new randomly generated values for interaction with the newly inserted gene. The stable vector also receives a new randomly generated value, representing the initial concentration of the product of the new gene. The result is a new individual with an extra transcription factor that may or may not be viable when developed with the augmented network.

**Figure 5:**
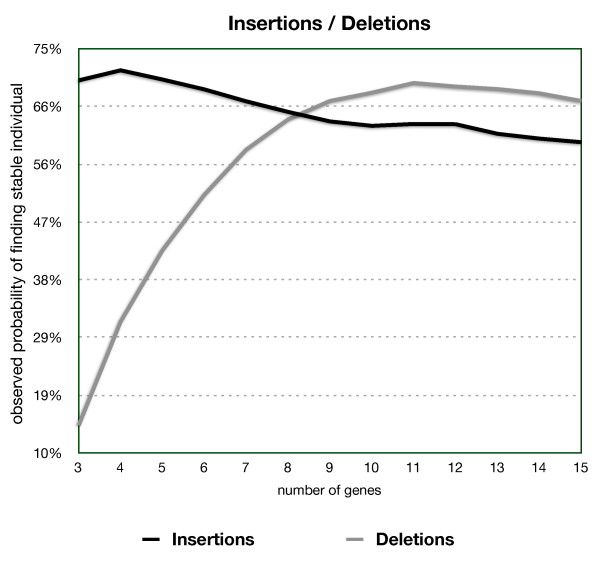
**Stability of new individuals generated through an insertion or a deletion**. The y-axis represents how many viable individuals a viable network with as many genes as represented by the x-axis generates after undergoing an insertion or a deletion.

From a population of 1000 viable networks we selected each in turn and tried 100 different random insertions and tested for stability. Each insertion adds a new gene at a random place in the network. The graph in Figure 5 shows how many of the 1000 networks yielded at least one viable individual after insertion. The number of genes shown in Figure 5 is the original number of genes in the network prior to the insertion. Insertions had a 62.6% success rate generating viable gene networks of 11 genes derived from 10-gene networks. The efficiency decreases as the number of genes increases, but still succeeds in 60.0% of the attempts of generating a viable network with 16 interacting genes.

**Figure 6:**
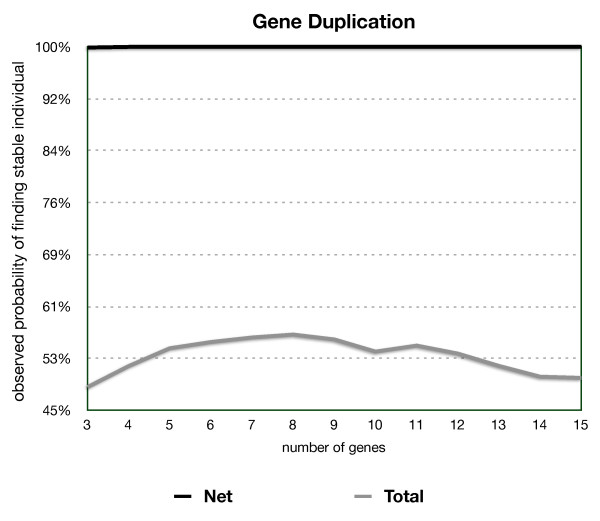
**As a special scenario of insertions, gene duplication consists of a full copy of a randomly selected row/column pair inserted into the individual's network resulting in an individual with an extra copy of a gene**. Gene duplication generates viable individuals around 50% of the time independent of the number of genes in the network. This independence of the number of genes in the network is a unique feature of gene duplication that allows creation of complex viable networks.

Figure 6 shows the result of duplicating an existing gene at random. In this case the probability of generating a viable network is about 50% independent of the number of genes in the network. Gene duplication therefore affords an efficient mechanism of increasing the dimensionality of viable gene networks.

### Random Deletion

Similarly to the test with random insertions, the likelihood of obtaining a viable network after removing a gene was tested by deleting one gene at random from a viable network and developing viable individual state vectors to asses if it would remain viable. We performed 100 random deletions in each of the 1000 previously generated viable networks. A gene deletion comprises a row and column deletion in the network, plus an entry deletion for the corresponding gene product in the initial output vector.

For networks with few interacting genes, loss of a gene is critical, with very few networks remaining viable after a deletion. This result is compatible with the difficulty in finding viable networks when there are few interacting genes. With more complex networks the numbers are still high, for example, 67.9% for networks with originally 10 interacting genes, which is significantly greater than the 14% rate for randomly generated networks with 9 interacting genes. Deletion maintains 66.6% of the viable networks with 15 interacting genes.

## Conclusion

We presented an alternative model to describe the development and evolution of gene transcription factors that allows for a continuous distribution of expression levels. This version of the model allows the study of more complex network (both in number of genes and degree of connectivity) given the additional classes of networks that yield viable individuals. The continuous model, however, makes it more difficult to define the concept of "neighboring networks" but this may be addressed by defining a threshold below which differences between networks define them as neighbors. Another limitation to our model is computing time, as the matrix multiplication in development and the tests for viability are more time consuming than in the discrete model.

## Authors' contributions

MC participated in the conception and design of the study and developed the computer simulations to perform the statistical analyses. CHT participated in the design and coordination and helped to draft the manuscript. DLH conceived the study and participated in its design and coordination and helped to draft the manuscript. All authors read and approved the final manuscript.

## Supplementary Material

Additional file 1 **Mathematical Background**. Formal mathematical background to the computations that are described in the body of this article and to compare the discrete model (step function) to the continuous model (ramp function). Click here for file
