# Supplementary material for: Model transcriptional networks with continuously varying expression levels
Source: BMC Evol Biol. 2011 Dec 19;11:363. doi: 10.1186/1471-2148-11-363 (PMC3270072; doi:10.1186/1471-2148-11-363)
Supplement: Additional file 1 — Mathematical Background. Formal mathematical background to the computations that are described in the body of this article and to compare the discrete model (step function) to the continuous model (ramp function). [file 1471-2148-11-363-S1.PDF]

Mathematical Background

Appendix

Our purpose here is to supply some mathematical background to the computations that are described in the body of this article and to compare the discrete model (step function) to the continuous model (ramp function).

1. The stochastic dynamics

Our dynamical system is defined for vectors in $\mathbb{R}^n$ with all entries between -1 and 1. This being the case, use Ω to denote this set of vectors. The k'th entry of a vector, v, is denoted by v_k and so $v \in \Omega$ if and only if $-1 \leq v_k \leq 1$ for all $k \in \{1, 2, \ldots, n\}$. If A is an $n \times n$ matrix, we use A_{ik} for $i, k \in \{1, \ldots, n\}$ to denote the entry in the i'th row and k'th column. Multiplication by the matrix A defines a linear transformation of $\mathbb{R}^n$ which sends any given vector v to the vector, $A\,v$, whose i'th component is $\sum_{k=1,2,\ldots n} A_{ik}v_k$. Our dynamical system on Ω also involves a filter function, f, this a function from $\mathbb{R}$ to the interval [-1, 1] which we assume is odd, thus $f(-x) = -f(x)$. The pair (A, f) is used to define the map $\mathbb{T}: \Omega \to \Omega$ by the rule that assigns any given vector $v \in \Omega$ the vector $\mathbb{T}_A(v)$ whose i'th component is

$$\mathbb{T}_A(v)_i = f(\Sigma_{k=1,\ldots,n} A_{ik}v_k). \tag{1.1}$$

We use $\mathcal{F}_A \subset \Omega$ to denote the set of stable fixed points of $\mathbb{T}_A$. Note that a fixed point said to be stable if the set of $k \in \{1, 2,\ldots\}$ fold itererates $\mathbb{T}_A(\mathbb{T}_A(\cdots(\mathbb{T}_A(\cdot))\cdots))$ of any vector in some small region around v defines a sequence of vectors in Ω that converges to the given fixed point. Sections 2-4 of what follows discuss $\mathbb{T}_A$ for various choices of the function f.

In Section 5, we view the assignment $A \to \mathcal{F}_A$ as a map from the space of $n \times n$ matrices to the sets in Ω, and are interested in the distribution and the properties of this map in a probablistic sense. This is to say that we fix a probability distribution, $\wp$, on $\mathbb{R}$, and use it to define a probability distribution on the space of $n \times n$ matrices whereby the n^2 entries are independent random variables with probability distribution $\wp$. This probability distribution on the space of $n \times n$ matrices is also denoted by $\wp$. The $\wp$-measure of a given subset of matrices is the probability as determined by $\wp$ of drawing a matrix from the subset.

Suppose that $\mathcal{F}$ is a given subset of Ω. We take $\wp$ to be a Gaussian distribution with mean zero and consider the corresponding $\wp$-measure of the set of $n \times n$ matrices A with $\mathcal{F}_A \cap \mathcal{F} \neq \emptyset$ for certain choices of f. The use of a Gaussian leads to relatively simple formula; and the results for a Gaussian indicate what happens in general. As a final topic

in Section 5, we take a function on Ω and use it to define a random variable on the space of matrices, this being its average over the fixed point set of the corresponding version of $\mathbb{T}_{(\cdot)}$. We give formulae for the corresponding mean and standard deviation in the case when $\wp$ is a Gaussian.

2. The fixed point set of $\mathbb{T}_{(\cdot)}$ and the function *f*

This section makes some simple observations that concern the function f. The next section considers some specific choices for f that define a corresponding version of $\mathbb{T}_{(\cdot)}$ whose fixed point set can be written explicitly for any given choice of matrix.

Fix a suitable function f and then an $n \times n$ matrix A. The origin, $0 \in \Omega$, is always a fixed point of $\mathbb{T}_{(A)}$.

Lemma 2.1: *Assume that f is differentiable at* $0 \in \mathbb{R}$. *The origin in* Ω *is a stable fixed point of f if and only if the absolute value of the derivative of f at* $0 \in \mathbb{R}$ *is less than the inverse of the largest of the absolute values of* A*'s eigenvalues.*

Proof: The transformation $\mathbb{T}_A$ near the origin can be written using Taylor's theorem as the map $v \to f_0{'}\, A v + \mathcal{O}(|v|^2)$ where $f_0{'}$ here denotes the derivative of f at 0.

The next lemma states a more general version of Lemma 2.1. The lemma assumes that f is differentiable on some interval around the origin. Assuming this is the case, introduce λ_f to denote the least upper bound for $\{|f(x)|/x\colon x \in (0, \infty)\}$.

Lemma 2.2: *Assume that f is differentiable near the origin. Then the origin is the only fixed point of* $\mathbb{T}_A$ *if* A *is such that* $|A v| < \lambda_f^{-1}|v|$.

Proof: If $\mathbb{T}_A(v) = v$, then $f(\sum_{k=1,\ldots,n} A_{ik} v_k) = v_i$ for all i. If $|f(x)| \le \lambda_f|x|$ for all x, then this requires that $|\sum_{k=1,\ldots,n} A_{ik} v_k| > \lambda_f^{-1}|v_i|$ for all i. But this is impossible if $|A v| < \lambda_f^{-1}|v|$.

Topological considerations can be invoked to infer the existence of an open set of matrices where the corresponding version of $\mathbb{T}$ has a non-zero fixed point. The next lemma states what may be the simplest one as the proof invokes only the notion of an *Euler number*. More sophisticated results can be derived by invoking, for example, Morse theory. We say nothing more about the latter and turn our focus instead on specific examples.

The lemma that follows uses $f'|_0$ to denote the derivative of f at the origin.

Lemma 2.3: *Suppose that f has a smooth inverse mapping* (-1, 1) *to* $\mathbb{R}$. *Let* A *denote a given matrix. There is a non-zero fixed point of* $\mathbb{T}_A$ *if* A *has the following two properties: First,* $(f'|_0)^{-1}$ *is not an eigenvalue of* A. *Second,* A *has an odd number of eigenvalues (counted with multiplicity) that are greater than* $(f'|_0)^{-1}$ *in the case when* $f'|_0 > 0$*; and it has an odd number that are less than* $(f'|_0)^{-1}$ *in the case when* $f'|_0 < 0$.

Proof: Let g denote the inverse function of f. This is to say that $g(f(v)) = v$. Let A denote a given matrix. The corresponding fixed point condition can be written as

$$(A v)_i - g(v_i) = 0.$$

What is written on the right hand side of this equality defines the components of a vector field on Ω. Assume first that $f'|_0 > 0$. Then the vector field in question points into Ω on the boundary since $x\, g(x) >> 1$ where $x \sim 1$. This being the case, its *Euler number* must be $(-1)^n$. If $f'|_0 < 0$, then the vector field points out along the boundary and the corresponding Euler number is +1.

To see what this implies, note that the vector field in question near the origin in Ω has components

$$(A v)_i - (f'|_0)^{-1} v_i \quad .$$

If $(f'|_0)^{-1}$ is not an eigenvalue of A, then the origin is a non-degenerate zero of the vector field; and so this zero contributes either +1 or -1 to a sum that defines the Euler number. The sign in question is that of the determinant of $(A - f'|_0^{-1}\mathbb{I})$ where $\mathbb{I}$ here denotes the identity matrix. If $f'|_0 > 0$ and this sign is $(-1)^n$, then there must be at least two other zeros of the relevant vector field, and thus two other fixed points of $\mathbb{T}_A$ in Ω. If $f'|_0 < 0$ and this sign is -1, then there must likewise be two other fixed points of $\mathbb{T}_A$.

If $f'|_0 > 0$ then this sign is $(-1)^n$ if and only if there are an odd number (counting multiplicity) of eigenvalue of A that are greater than $(f'|_0)^{-1}$. If $f'_0 < 0$, then this sign equal to -1 if and only if A has an odd number of eigenvalues (counting multiplicity) that are less than $(f'|_0)^{-1}$.

3. The ramp function

This section considers the special case where $f(x)$ is given by the rules

$$f(x) = -1 \ \textit{if}\ x \le -1, \quad f(x) = x \ \textit{if}\ -1 \le x \le 1 \quad , \quad f(x) = 1 \ \textit{if}\ x \ge 1. \tag{3.1}$$

The first lemma tells us where to look for non-zero fixed points.

Lemma 3.1: *Let* A *denote a matrix with no eigenvalue equal to 1. All non-zero fixed points of* $\mathbb{T}_A$ *are on the boundary of* Ω.

Proof: If v is not on the boundary of Ω, then $\mathbb{T}_A(v) = A \cdot v$ and so either v = 0 or 1 is an eigenvalue of A.

Fix k ∈ {1, …, n}. A k-facet of Ω consists of the set of points on the boundary of Ω where precisely k of the entries have absolute value equal to 1. For example, if n = 3, then Ω is a cube in $\mathbb{R}^3$ and a 1-facet is a face, a 2-facet is an edge, and a 3-facet is a vertex. In any event, there are $\frac{n!}{k!(n-k)!} 2^k$ different k-facets.

Let A denote a given n × n matrix. Fix k ∈ {1, …, n}. What follows describes the fixed points of $\mathbb{T}_A$ on the k-facet where the first k entries are 1. The story for the other k-facets can be obtained from what is said below by relabling indices and reversing some signs. The story for these other facets is summarized by Lemma 3.2.

To describe the fixed points on the facet with first k-entries equal to 1, it proves useful to write $\mathbb{R}^n = \mathbb{R}^k \times \mathbb{R}^{n-k}$ and write a given vector v in a corresponding 2-component fashion as

$$v = \begin{pmatrix} u \\ w \end{pmatrix}, \tag{3.2}$$

where $u \in \mathbb{R}^k$ and $w \in \mathbb{R}^{n-k}$. We use ı in what follows to denote the vector in $\mathbb{R}^k$ with all entries equal to 1. Thus, v is in the relevant k-facet if and only if a = ı. The matrix A is written in block diagonal form as

$$A = \begin{pmatrix} \alpha & \beta \\ \gamma & \Delta \end{pmatrix}, \tag{3.3}$$

where α is a k × k matrix, β is linear transformation from $\mathbb{R}^{n-k}$ to $\mathbb{R}^k$ (thus a matrix with k rows and n - k columns), γ is a linear transformation from $\mathbb{R}^k$ to $\mathbb{R}^{n-k}$ (a matrix with n - k rows and k columns), and Δ is an (n-k) × (n - k) matrix.

Granted this notation, then $v = \begin{pmatrix} ı \\ w \end{pmatrix}$ is a fixed point of $\mathbb{T}_A$ if and only if the following two conditions are met:

- $(\alpha\, ı + \beta w)_i > 1$ *for all* i ∈ {1, …, k}.
- $|w_x| < 1$ *for all* x ∈ {1, …, n-k}.

- $w = \gamma I + \Delta w$.

$$(3.4)$$

If 1 is not an eigenvalue of Δ, then the third bullet in (3.4) tells us that $w = (1 - \Delta)^{-1}\gamma I$, the second bullet says that the vector $(1 - \Delta)^{-1}\gamma I$ has all entries with absolute value less than 1, and the first bullet tells us that the sum of the entries in each row of the $k \times k$ matrix

$$\alpha + \beta(1 - \Delta)^{-1}\gamma \tag{3.5}$$

must be at least 1. The fixed point in question is stable if the sum of the entries of each row of (3.5) is strictly greater than 1. If Δ has eigenvalue 1, then there is a fixed point on this facet if and only if γI is orthogonal to the eigenvector of the transpose of Δ with eigenvalue 1 and also the first two bullets of (3.4) hold. The corresponding fixed point will not be stable.

Any given facet defines a corresponding decomposition of the matrix A and so corresponding versions of α, β, γ and Δ. There is also a corresponding version of the vector I, and a corresponding version of (3.4). To say something precise, remark that the facets are in 1-1 correspondence with the set of diagonal matrices with at least one non-zero entry and all entries either +1, -1 or zero. A matrix of this sort is said to be a *facet* matrix. For example, the facet matrix for the facet whose vectors have first k entries equal to 1 is the diagonal matrix with the first k diagonals equal to 1. Let D denote a facet matrix with k entries non-zero. We view D as a linear transformation from $\mathbb{R}^n$ to $\mathbb{R}^k$. We identify the kernel of D with $\mathbb{R}^{n-k}$ and use Π_D to denote the orthogonal projection from $\mathbb{R}^n$ to this version of $\mathbb{R}^{n-k}$. The corresponding versions of α, β, γ, and Δ are the given respectively by $\alpha_D = D A D^T$, $\beta_D = D A \Pi_D^T$, $\gamma_D = \Pi_D A D^T$, and $\Delta_D = \Pi_D A \Pi_D^T$. The corresponding version of the vector I is obtained by applying D to the vector in $\mathbb{R}^n$ with all entries equal to 1. This vector is denoted by I_D.

Lemma 3.2: *Let* A *denote a given matrix and let* D *denote a given facet matrix. Then* $\mathbb{T}_A$ *has at most one stable fixed point on the corresponding facet and this fixed point exists if and only if the following conditions are met:*

- *The matrix* Δ_D *lacks* 1 *as an eigenvalue.*
- *The vector* $(1 - \Delta_D)^{-1}\gamma_D I_D$ *has all entries with absolute value less than 1.*
- *Corresponding entries of the vectors* I_D *and* $(\alpha_D + \beta_D(1 - \Delta_D)^{-1}\gamma_D) I_D$ *have the same sign, and those of the latter have greater absolute value.*

Proof: Acting on $\mathbb{R}^n$ by a suitable orthogonal matrix that switches entries and multiplies some entries by (-1) takes D to a facet matrix of the sort that corresponds to some k-facet where the vectors have first k entries equal to 1. This understood, the assertion of the

lemma is obtained by applying thte inverse of this orthogonal transformation to reinterpret (3.4).

4. The step function

Fix $t \in [0, 1]$. We consider here the following version of f:

$$f(x) = -1 \text{ if } x < -t, \quad f(x) = 0 \text{ if } -t \le x \le t, \quad f(x) = 1 \text{ if } x > t\,. \tag{4.1}$$

Suppose that A is a given matrix. The following lemma describes the fixed point of $\mathbb{T}_A$.

Lemma 4.1: *Let* A *denote a given non-zero matrix. The origin is a stable fixed point of* $\mathbb{T}_A$ *if and only if* $t > 0$. *In any case, suppose that* D *is a given facet matrix. Then there is at most 1 stable fixed point of* $\mathbb{T}_A$ *on the corresponding facet, and such a fixed point exists if and only if the following conditions hold*:

- *All entries of the vector* $\gamma_D I_D$ *have absolute value less than* t.
- *Corresponding entries of the vectors* I_D *and* $\alpha_D I_D$ *have the same sign and the latter have absolute value greater than* t.

The entries of this stable fixed point are the diagonal entries of D.

Note that in the case when $t = 0$, then Lemma 4.1 asserts that the only the vertices of the hypercube Ω can be stable fixed points.

Proof: The all entries of $\mathbb{T}_A(v)$ are ± 1 or 0 for any given vector v. As a consequence, the fixed points must have all entries either ± 1 or zero. If $t = 0$, then $\mathbb{T}_A(v) = 0$ if and only if $v = 0$, so 0 is not a stable fixed point. On the other hand, if $t > 0$, then $\mathbb{T}_A(v) = 0$ if $|v|$ is sufficiently small because all entries of Av when $|v|$ is small will have absolute value less than t. Thus, if $t > 0$, then the origin is a stable fixed point.

As in the case of Lemma 3.2, it is sufficient to consider only fixed points on those k-facets where the first k entries are equal to 1. Given such a facet, reintroduce the decomposition of $\mathbb{R}^n$ as depicted in (3.2) and the corresponding block diagonal decomposition of A as depicted in (3.3). A fixed point on this strata can only be the vector

$$\begin{pmatrix} I \\ O \end{pmatrix} \tag{4.2}$$

where $O \in \mathbb{R}^{n-k}$ is the origin. Moreover, this vector is a stable fixed point if and only if

- *Each entry of* $\alpha\iota$ *is greater than* t.
- *Each entry of* $\gamma\iota$ *has absolute value less than* t.

(4.3)

There is an unstable fixed point on this facet if one of the inequalities in (4.3) is a strict equality. By way of elaboration, these last observations follow because A maps the vector in (4.2) to the vector $\begin{pmatrix} \alpha\iota \\ \gamma\iota \end{pmatrix}$.

5. Stochastics

This section discusses the stochastic aspects of the step function model given in the previous section. An analysis of the ramp model can be made along the same lines. In any event, the step function model nicely illustrates the fundamental issues.

The simplest case to consider is the t = 0 step function model.

Lemma 5.1: *Assume that the probability distribution* $\wp$ *is given by a smooth function. In the case of the* t = 0 *step function model, there is a* $\wp$*-measure* 1 *subset of matrices for which the set of vertices of* Ω *is the set of stable fixed points of the corresponding version of* $\mathbb{T}_{(\cdot)}$.

Note that this lemma asserts that the model with t = 0 is completely insensitive to the choice for the probability distribution $\wp$.

Proof: This follows directly from Lemma 4.1 as the latter's conditions are violated only by matrices that lie in some subspace with positive codimension in the space of $n \times n$ matrices.

We now consider a given t > 0 step function model; and we take $\wp$ to be the Gaussian distribution with mean 0 and standard deviation $\sigma > 0$. This is to say that

$$\wp(x) = \frac{1}{\sigma\sqrt{2\pi}}\, e^{-x^2/(2\sigma^2)}. \tag{5.1}$$

A corresponding analysis can be done for any given probability distribution.

To set the stage for what is to come, remark that the $\sigma = 1$ version of $\wp$ is the Gaussian normal distribution. Let $x \to E[x]$ denote the integral of the normal distribution over the interval [0, x]. The integral of (5.2) over this same interval [0, x] is $E[x/\sigma]$.

Lemma 5.1: *Fix* $t > 0$ *to define the step function model and take* $\wp$ *to be a given* $\sigma > 0$ *version of (5.1). The set of matrices with* $0 \in \Omega$ *as a stable fixed point of* $\mathbb{T}_{(\cdot)}$ *has* $\wp$*-measure 1. The set of matrices with a given non-zero vector in* Ω *as a stable fixed point of* $\mathbb{T}_{(\cdot)}$ *is zero unless the vector is the shortest vector on some* k*-facet. In the latter case, the set of matrices in question has* $\wp$*-measure*

$$(1 - E[\sqrt{k}\, t/\sigma])^k \, E[\sqrt{n-k}\, t/\sigma]^{n-k}.$$

Proof: This follows directly from the conditions given by Lemma 4.1 given the following additional observation: Fix $m \in \{1, 2, \ldots\}$ and let $\{x_a\}_{a=1,2,\ldots m}$ denote a set of m independent random variables, all with the normal probability distribution. Let $x = \sum_{a=1,\ldots m} x_a$ denote the sum of these random variables. Then the induced probability distribution of x is the Gaussian distribution with mean 0 and $\sigma = m^{-1/2}$.

Fix $t \in [0, 1]$ to specify the relevant step function model. Any given matrix A has a corresponding subset $\mathcal{F}_A$ of stable fixed points for the corresponding version of $\mathbb{T}_A$. This set contains the origin and some subset of the set of shortest vectors on the various facets of Ω. (There are $3^n - 1$ of the latter.) Given $k \in \{1, \ldots, n\}$, we use Λ_k to denote the set of shortest vectors on the various k-facets. This set has $\frac{n!}{k!(n-k)!} 2^k$ elements.

Let $h: \Omega \to \mathbb{R}$ denote a given function, and define

$$ph_A = \sum_{v \in \mathcal{F}_A} h(v) . \tag{5.2}$$

The assignment $A \to ph_A$ defines a random variable on the space of $n \times n$ matrices. With $\sigma > 0$ chosen to define $\wp$ via (5.1), we use μ_{ph} to denote the mean of the random variable $A \to ph_A$ and we use σ_{ph} to denote the standard deviation. If $t = 0$, then Lemma 5.1 finds $\mu_{ph} = \sum_{v \in \Lambda_n} h(v)$ and $\sigma_{ph} = \sum_{v \in \Lambda_n} h(v)^2 - \mu_{ph}^2$. When $t > 0$, the Lemma 5.1 finds

- $\mu_{ph} = h(0) + \sum_{k=1,\ldots,n}\{(1 - E[\sqrt{k}\, t/\sigma])^k \, E[\sqrt{n-k}\, t/\sigma]^{n-k} \langle h\rangle_k\}$.
- $\sigma_{ph}^2 = h(0)^2 + \sum_{k=1,\ldots,n}\{(1 - E[\sqrt{k}\, t/\sigma])^k \, E[\sqrt{n-k}\, t/\sigma]^{n-k} \langle h^2\rangle_k\} - \mu_{ph}^2$.

(5.3)

where $\langle h\rangle_k = \sum_{v \in \Lambda_k} h(v)$ and $\langle h^2\rangle_k = \sum_{v \in \Lambda_k} h(v)^2$.

By way of an example, if $p \in \{1, 2, \ldots\}$ and $h(v) = \sum_{i=1,\ldots,k} v_i^p$, then h(v) is zero if p is odd, and $h(v) = k$ if $v \in \Lambda_k$. In the latter case $\langle h_k\rangle = \frac{n!}{(k-1)!(n-k)!}$ and $\langle h^2\rangle_k = \frac{n!}{(k-2)!(n-k)!}$.

By way of a second example, consider the simplest sort of 'switch'; this is defined by taking $h(v) = 1$ if $v_1 > s$ and $h(v) = 0$ if $v_1 \le s$ where $s \in [0, 1)$ is a given number. In this case, $\langle h\rangle_k = \langle h^2\rangle_k = \frac{n!}{(k-1)!(n-k)!}$.
